# Rapamycin up-regulation of autophagy reduces infarct size and improves outcomes in both permanent MCAL, and embolic MCAO, murine models of stroke

**DOI:** 10.1186/2040-7378-6-8

**Published:** 2014-06-21

**Authors:** Kathleen M Buckley, Daniel L Hess, Irina Y Sazonova, Sudharsan Periyasamy-Thandavan, John R Barrett, Russell Kirks, Harrison Grace, Galina Kondrikova, Maribeth H Johnson, David C Hess, Patricia V Schoenlein, Md Nasrul Hoda, William D Hill

**Affiliations:** 1 Charlie Norwood VA Medical Center, Augusta, GA, USA; 2 Department of Cellular Biology & Anatomy, Georgia Regents University, Augusta, GA, USA; 3 The University of Virginia, School of Medicine, Charlottesville, VA, USA; 4 Department of Neurology, Georgia Regents University, Augusta, GA, USA; 5 Department of Medicine, Georgia Regents University, Augusta, GA, USA; 6 Department of Emergency Medicine, The University of Pennsylvania, Philadelphia, PA, USA; 7 Department of Surgery, Carolinas Medical Center, Charlotte, NC, USA; 8 Medical College of Georgia, Georgia Regents University, Augusta, GA, USA; 9 Department of Biostatistics and Epidemiology, Georgia Regents University, Augusta, GA, USA; 10 Medical Laboratory, Imaging & Radiologic Sciences, Georgia Regents University, Augusta, GA, USA; 11 Program in Clinical and Experimental Therapeutics, College of Pharmacy, University of Georgia, Augusta, GA, USA

**Keywords:** Cerebral ischemia, Embolic stroke, Autophagy, Rapamycin, Chloroquine

## Abstract

**Background and purpose:**

The role of autophagy in response to ischemic stroke has been confusing with reports that both enhancement and inhibition of autophagy decrease infarct size and improve post-stroke outcomes. We sought to clarify this by comparing pharmacologic modulation of autophagy in two clinically relevant murine models of stroke.

**Methods:**

We used rapamycin to induce autophagy, and chloroquine to block completion of autophagy, by treating mice immediately after stroke and at 24 hours post-stroke in two different models; permanent Middle Cerebral Artery Ligation (MCAL), which does not allow for reperfusion of distal trunk of middle cerebral artery, and Embolic Clot Middle Cerebral Artery Occlusion (eMCAO) which allows for a slow reperfusion similar to that seen in most human stroke patients. Outcome measures at 48 hours post-stroke included infarct size analysis, behavioral assessment using Bederson neurological scoring, and survival.

**Results:**

Chloroquine treatment reduced the lesion size by approximately 30% and was significant only in the eMCAO model, where it also improved the neurological score, but did not increase survival. Rapamycin reduced lesion size by 44% and 50% in the MCAL and eMCAO models, respectively. Rapamycin also improved the neurological score to a greater degree than chloroquine and improved survival.

**Conclusions:**

While both inhibition and enhancement of autophagy by pharmacological intervention decreased lesion size and improved neurological scores, the enhancement with rapamycin showed a greater degree of improvement in outcomes as well as in survival. The protective action seen with chloroquine may be in part due to off-target effects on apoptosis separate from blocking lysosomal activity in autophagy. We conclude pharmacologic induction of autophagy is more advantageous than its blockade in physiologically-relevant permanent and slow reperfusion stroke models.

## Introduction

Autophagy, a cell survival process has recently been recognized as being involved in human strokes [1,2]. The major form of autophagy, macroautophagy (referred to as autophagy herein), is a cellular process that normally recycles old, or damaged, organelles and proteins by vesicular seclusion followed by lysosomal degradation. It helps in reclaiming essential nutrients and molecular components and can be an alternative source of ATP. It is up regulated by cellular stressors, including nutrient deprivation, ischemia and reactive oxygen species (ROS) to enhance cell survival. In that many of these same stressors occur in ischemic stroke, it is of interest to understand the role of autophagy following stroke [1,3,4]. Autophagy has been implicated in recovery from cardiac ischemia [5], and also retards the progression of chronic neurodegeneration [6]. Autophagy dysregulation may underlie a number of CNS disorders. Normal regulation of protective autophagy may be overwhelmed following large ischemic injuries, which either allow apoptotic cell death to precede, or even lead to an autophagic mediated form of cell death [7]. As such, it is controversial whether increasing, or inhibiting, autophagy is protective following stroke. While autophagy has been studied in animal models of adult and neonatal brain ischemia, most of the studies are inconclusive, or have produced contradictory outcomes [1,3,4,7]. This lack of consensus on the role of autophagy in stroke injury may in large part be due to methodological approaches where autophagy has been either “induced” or “inhibited”, but not compared side by side in the same study. Confounding the issue, some of the autophagy inhibitors used can also inhibit apoptotic pathways, or can even increase autophagy with prolonged treatment [8].

The type of ischemic models used, in particular “permanent ischemia” or slow reperfusion vs rapid reperfusion models, may also impact the outcome of manipulating autophagy pathways as has been reported in some myocardial infarction studies [9]. Importantly, the use of pre-clinical animal stroke models that more closely and effectively model key physiologic mechanisms involved in human stroke has been highlighted as critical to success in therapeutic human clinical trials [10,11]. To date there has been almost universal failure of neuroprotection based clinical trials following successful pre-clinical animal studies.

Following ischemic cerebral stroke two major vascular pathophysiological processes impact stroke outcomes and should be considered in pre-clinical models to more effectively mimic human stroke injury. The first is the severity of the cerebral blood flow (CBF) reduction and the second is the dynamics of CBF restoration [11]. The severity of CBF reduction is critical in human stroke and several pre-clinical rodent models effectively address this aspect. However, dynamics of CBF restoration are frequently not modeled closely. Rapid complete reperfusion where the timing of the reperfusion is prior to irreversible ischemic core damage and penumbral tissue necrosis, is quite different from the type of reperfusion seen in human stroke either with, or without, tPA treatment or surgical thromboectomy. Indeed even timely tPA or thromboectomy treatments, which are available to less than 5 percent of all patients, have significantly different reperfusion characteristics than most animal models of stroke [12]. As such the most commonly used rodent models, including transient suture occlusion of the middle cerebral artery (tsMCAO), result in non-physiologic rapid reperfusion. Early, rapid reperfusion involves significant differences in a number of aspects including: vascular dynamics (e.g. degree of reperfusion, reperfusion pressure, blood viscosity, vessel dilation, no reflow) and endothelial cell responses, reactive oxygen species production, downstream micro-infarction from degraded embolic clots, and bioactive clot derived molecules [10,11]. Full restoration of cerebral blood flow within 3–6 hours, depending on the initial size of the ischemic region, can potentially preserve penumbral tissue outside the ischemic core, though tissue recovery within the ischemic core requires an even shorter time window, and this penumbral tissue is then vulnerable to secondary cell death mechanisms [10,11]. However, within these time frames most human cases remain either non-reperfused, or are only partially reperfused in a delayed fashion, due to limited self-recanalization [10,11]. Therefore, to more closely replicate human CBF dynamics two different stroke models were used here. First, a middle cerebral artery ligation (MCAL) model was used that produces a permanent occlusion with similarities to small non-reperfused human stroke [11]. Second, we also included a more physiologically relevant murine embolic clot middle cerebral artery occlusion (eMCAO) model [13,14]. This model uses embolic clots prepared from murine blood stabilized with human fibrinogen to permit slow self-recanalization over the course of 24-plus hours.

In this study, we examined the effect of pharmacologic modulation of autophagy as a treatment strategy to protect brain tissue from nutrient and energy deficits with delayed reperfusion [11,13]. Given the confusion of scientific literature on the role of autophagy, in this initial study we used two pharmacological agents to drive (rapamycin), or block (chloroquine), autophagy. We tested the hypothesis that inducing autophagy during ischemia in permanent and slow partial reperfusion stroke models is advantageous over its blockade in decreasing post-stroke injury and in improving outcomes.

## Materials and methods

### Animals

C57BL/6 J male mice were purchased from Jackson Laboratories (Bar Harbor, ME). Animals were maintained at the Animal Research Facility of the Charlie Norwood Veterans Affairs Medical Center (VAMC) with free access to chow diet, water *ad*-*libitum* and 12 hours light/dark cycle. We used either 8–12 or 20–22 weeks old mice for MCAL or eMCAO, respectively. We chose to use young mice in the MCAL model experiments based on our extensive preliminary studies establishing dose and timing of rapamycin and chloroquine treatments [13,14]. However, we used older mice with the eMCAO model based on our experience with the development of that model and increased physiologic relevancy of using older mice [13-15]. Experiments were conducted in accordance with the guidelines set by the Institutional Animal Care and Use Committees (IACUC) of the Charlie Norwood VAMC and Georgia Regents University.

### Immunoblotting

Tissues were homogenized and lysed in 2× Laemmli lysis buffer, as previously described [16]. Membranes were re-probed with mouse monoclonal anti-GAPDH (G8795, Sigma-Aldrich Co.) and β-actin (A5441, Sigma-Aldrich Co.) as loading controls. All loading was based on EZQ™ Protein Quantitation Kit (Life Technologies) for equal loading. The two loading controls were used because we were looking at different tissues with very different cellular and extracellular protein expression. β-actin has proven to be useful for most tissues as a house-keeping gene, however it is not well expressed in muscle tissues. Consequently we also used GAPDH as a house-keeping control, which is expressed in muscle. However, its expression can vary across different tissues as well. Therefore, we included both loading controls. Proteins were visualized by ECL (Pierce, Thermo Fisher Scientific) using autoradiography film (Denville Scientific). The intensity of the immunoreactive bands was quantified by densitometry using Image J 1.44p software (NIH). All quantitations were performed off film exposed for one minute with equal antibody concentrations used on each tissue.

### Middle cerebral artery ligation (MCAL) stroke model

The MCAL surgery was performed as described previously with minor modifications [11]. A 2 – 3 mm burr hole was drilled at the junction of the zygomatic arch and the squamous bone. The main trunk of left MCA was exposed and electrocauterized distal to the lenticulostriate branches. The treatments were injected intra-peritoneal (IP) immediately after occlusion and repeated at 24 hours post-stroke. Animals were euthanized and tissue collected at 48 hours post-stroke as previously described [13].

### Embolic middle cerebral artery occlusion (eMCAO) stroke model

The murine eMCAO stroke model, using human fibrinogen (hu-fibrinogen) to stabilize the clot, was performed as recently reported [13,14]. Our eMCAO model was modified with human fibrinogen to improve stability and reproducibility [13,14]. Subsequently, other groups have also published that the addition of fibrinogen increases reproducibility in different rodent species. During the standardization of the embolic model using partially humanized clots, we studied the immune responses to injury either immunizing the mouse with higher (1 mg/kg) hu-fibrinogen or by released with clot lysate generated by using tPA/plasminogen thrombolysis. There were no significant differences in stroke outcomes from controls (data not shown). Additionally, no xenogenic immune effects have been reported by other laboratories [17-19]. The treatments and euthanasia were performed as described for MCAL.

### Pharmacological treatments

Normal control (no surgery, no treatment); vehicle control (either of the stroke surgeries followed by IP 40% DMSO for rapamycin in initial studies, or saline for chloroquine and follow up studies, treatment); rapamycin (Selleck, S1039; 0.625, 1.25, 2.5 mg/kg prepared in 4:10 DMSO:saline); chloroquine (Sigma-Aldrich Co., C6628; 30, 60, 90 mg/kg prepared in saline) groups were used (Additional file 1: Figure S1).

### Neurological assessment

Neurological deficits in the animals were assessed at 48 hours post-stroke using a modified 5-point Bederson scale, as described in detail previously [13,14]. 0, no deficit; 1, forelimb flexion deficit on contralateral side; 2, decreased resistance to lateral push and torso turning to the ipsilateral side when held by tail; 3, very significant circling to affected side and reduced capability to bear weight on the affected side; 4, animal rarely moves spontaneously and prefers to lay down or stay at rest.

### Infarct analysis

Infarct analysis was performed on 2,3,5-triphenyltetrazolium chloride (TTC; Sigma-Aldrich Co.) stained sections as described previously [13,14]. Brains were perfused with ice cold 0.01 M phosphate-buffered saline (PBS), cut into 1-mm coronal slices, stained by TTC for 30 minutes at 37°C, then fixed with 10% formalin in PBS. The images were digitalized and the infarct volume was analyzed by Image J software outlining the ipsilateral and contralateral hemispheres, excluding the infarcted tissue and including the infarcted region (if the fragile ischemic tissue fell out of the slice the original contour over that small area was derived from the corresponding contralateral zone after inversion. The infarct volumes were quantified as both direct volume (in mm^3^) and indirect volume (percent volume of the total ischemic hemisphere). An investigator blinded to treatment groups analyzed the infarct size.

### Autophagic flux

To assess autophagic flux *in vivo*, 22 week old mice were treated with 60 mg/kg chloroquine IP for five to six hours (n = 4) and compared to aged-matched controls receiving saline (n = 5). Mice were sacrificed by CO_2_ asphyxiation followed by thoracotomy. Tissues (Brain, lungs, heart, quadriceps muscle, spleen, kidney, and liver) were dissected and immediately snap-frozen in liquid nitrogen. The time and potential methods for autophagic flux assessment using inhibition of flux by chloroquine was modified from Klionsky et al. [20]. Tissues were processed for immunoblotting as described above.

### Statistical analyses

All the data are expressed as mean ± SD. Assumptions of normality were tested and rank transforamations were used as needed. Data were analyzed using SAS 9.3 software (SAS Institute Inc., Cary, NC). Two-sample t-tests were used to compare LC3 (I and II) quantitation of non-muscle, muscle and CQ treated brain tissue to non-treated brain tissue. A one-way analysis of variance (ANOVA) was used to determine dose effects for rapamycin or chloroquine. Infarct volume and neurological deficit were analyzed using a 2 rapamycin (none vs. 1.25 mg/kg) by 2 chloroquine (none vs. 60 mg/kg) ANOVA. An interaction was tested and if significant would indicate a differential effect of rapamycin in the presence of chloroquine. Tukey’s post-hoc tests were to adjust for multiple comparisons for significant ANOVA effects. Survival at 48 hours was analyzed using a Cochran-Mantel-Haenszel test. Null hypotheses were rejected at p ≤ 0.05.

## Results

### Autophagy in the brain

**Figure 1:**
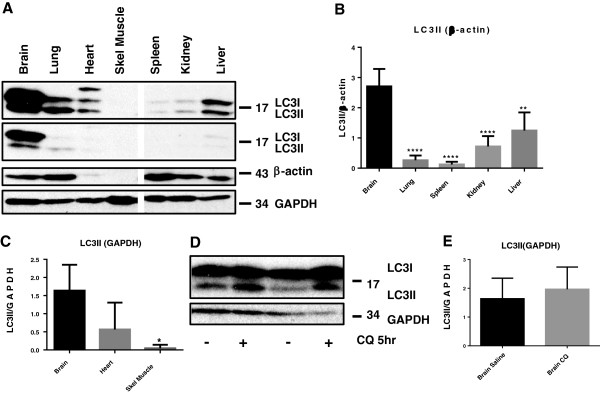
**Brain has the highest level of autophagy relative to other tissues. A)**. Brain, lung, heart, skeletal muscle, spleen, kidney and liver were collected from non-stroked 22–24 week old male C57BL/6 J mice. The tissues were assessed for the autophagy markers, LC3 I and II, and the loading control β-actin for non-muscle tissues and brain and GAPDH for muscle tissues and brain. The top subpanel in **(A)** is a 5-fold longer exposure than the subpanel immediately below it to highlight LC3I/II in tissues that were below the level of detection in the lower subpanel. The lower subpanel (exposed for 1 minute) shows the expression of brain LC3I/II relative to other tissues better, and with a better dynamic range, without the loss of linearity seen with the longer exposure. All subpanels in **(A)** are from the same blot, one tissue not used in other blots assessed in panels **B** &**C** was cropped out. A white space was left to show where the cropping occurred. **B)**. Quantitation of non-muscle tissues normalized to β-actin and compared to brain. All quantitations were performed on film exposed for one minute, with equal antibody concentrations and incubation used on each tissue set. **C)**. Quantitation of muscle tissues and brain normalized to GAPDH and compared to brain. **D)**. Two saline treated and two chloroquine treated brain samples are shown as representative examples. The chloroquine treated mice show apparent accumulation of LC3II following chloroquine treatment (five hours with 60 mg/kg chloroquine) to assess autophagic flux. **E)**. LC3II levels in brain samples from four chloroquine treated and five saline treated mice were measured. The accumulation of LC3II is noticeable, but not significantly different when quantitated against GAPDH. (* = p < 0.05, ** = p < 0.01, *** = p < 0.005, **** = p < 0.0001).

We demonstrated that the brain has the highest level of autophagy relative to other metabolically active tissues. The basal level of LC3 (microtubule-associated protein 1 light chain 3) isoforms, key markers to assess autophagy, was measured in different tissues from non-stroked mice to compare active autophagy (i.e. autophagic flux) across tissues [20,21]. Specifically, we compared levels of the most commonly used markers for autophagy, LC3I and II, in brain, heart, lungs, liver, kidney, spleen, and skeletal muscle from 22–24 week old male C57BL6 mice (Figure 1A-C). Additionally, we found a trend for LC3II to accumulate following brief chloroquine treatment (Figure 1D-E). Mice were treated with saline, or with 60 mg/kg chloroquine IP 4–6 hours prior to sacrifice to block the final steps of autophagy allowing accumulation of proteins that are normally degraded in active autophagy thereby permitting quantification of autophagic flux. Short-term treatment with chloroquine inhibits lysosomal enzyme activity in autolysosomes preventing the turnover of contents accrued within autophagosomes. The brain has both the high potential for constitutive autophagy, i.e. it is primed with high levels of LC3I, and has an actual high level of constitutive autophagic flux, or active autophagy with high LC3II levels that appear to increase with brief inhibition of autophagosome turnover.

### Modulation of autophagy in MCAL stroke model

**Figure 2:**
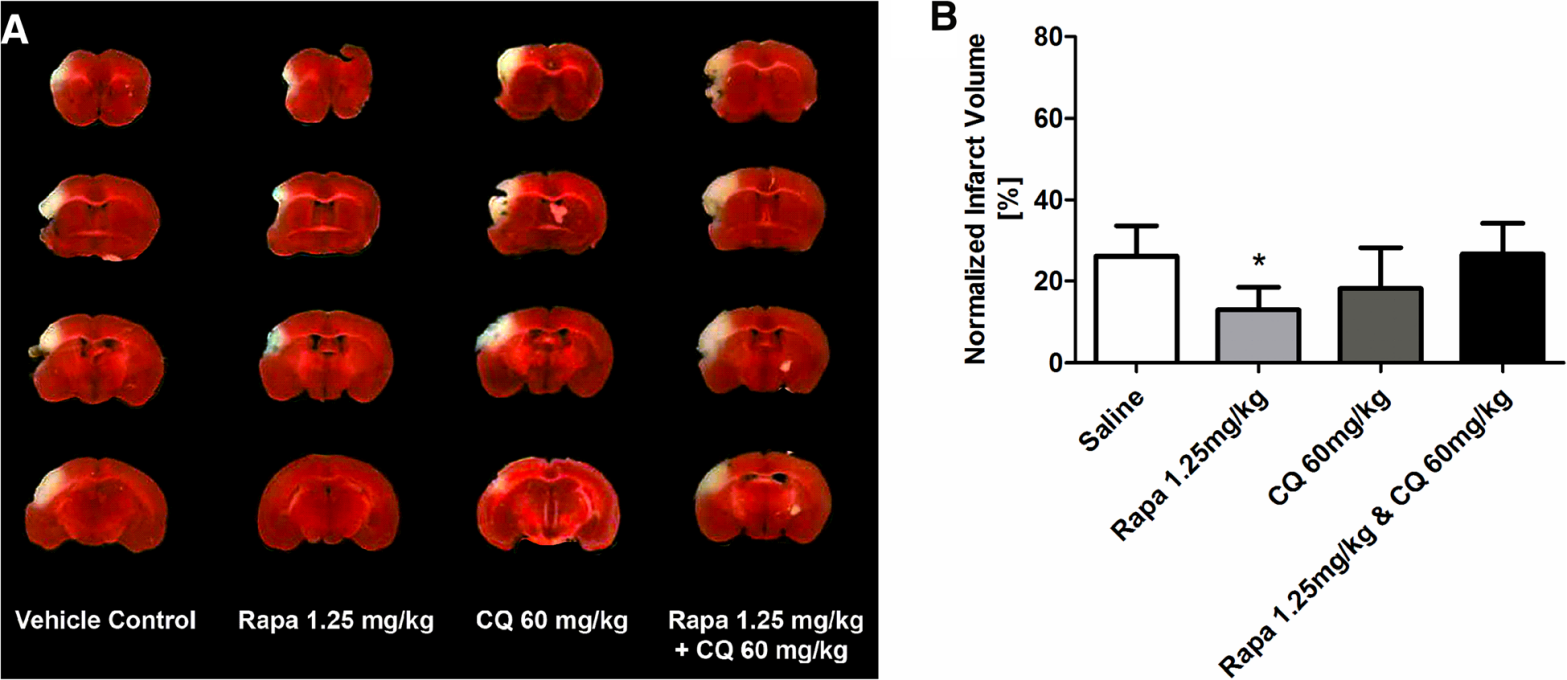
**Enhancement of autophagy with rapamycin decreases infarct size in MCAL mouse models of ischemic stroke injury. A)**. 8–12 week old male C57BL/6 J mice underwent MCAL stroke injury, and were treated twice (0 and 24 hours) with either saline, rapamycin (rapa) to enhance autophagy, chloroquine (CQ) to inhibit autophagy, or a combination of rapa and CQ. Infarct size analysis using 2% TTC staining (n = 9 mice per group). **B)**. Quantitation of panel **A** data (* = p < 0.05). Note tissue drop out in the lesion site is due to the fragility of the tissue, not surgical damage.

Initial studies using the MCAL stroke model were performed to assess the optimal doses of chloroquine (30, 60 and 90 mg/kg) and rapamycin (0.625, 1.25, 2.5 mg/kg) to use in further experiments (Additional file 1: Figure S1). The doses of 60 mg/kg for chloroquine and 1.25 mg/kg for rapamycin were determined to be the most efficacious in limiting lesion size and to minimize non-autophagic effects, and there was no difference in either the saline or DMSO controls relative to non-stroked mice. Infarct analyses by TTC staining after MCAL revealed that only rapamycin treatment significantly reduced the stroke lesion size as compared to the saline control group (50.8% relative reduction; 12.9% ±5.5% versus 26.1% ±9.1%, P < 0.05) (Figure 2). Chloroquine treatment also appears to reduce the infarct size, but not significantly, compared to saline controls (29.1% relative reduction; 18.5% ±10.1% versus 26.1% ±9.1%, P = 0.36). The significant neuroprotection conferred by rapamycin vanished when combined with the autophagy inhibitor chloroquine (26.6% ±7.6% versus 26.1% ±9.1%, P = 0.9). This is consistent with the two agents driving autophagy in opposite directions and that together they dysregulate autophagy. It also supports the idea that rapamycin’s major effect is through induction of autophagy rather than through other mechanisms, e.g. immunosuppression, since there were no synergistic, or additive interactions with the combined treatments.

### Modulation of autophagy in eMCAO stroke model

**Figure 3:**
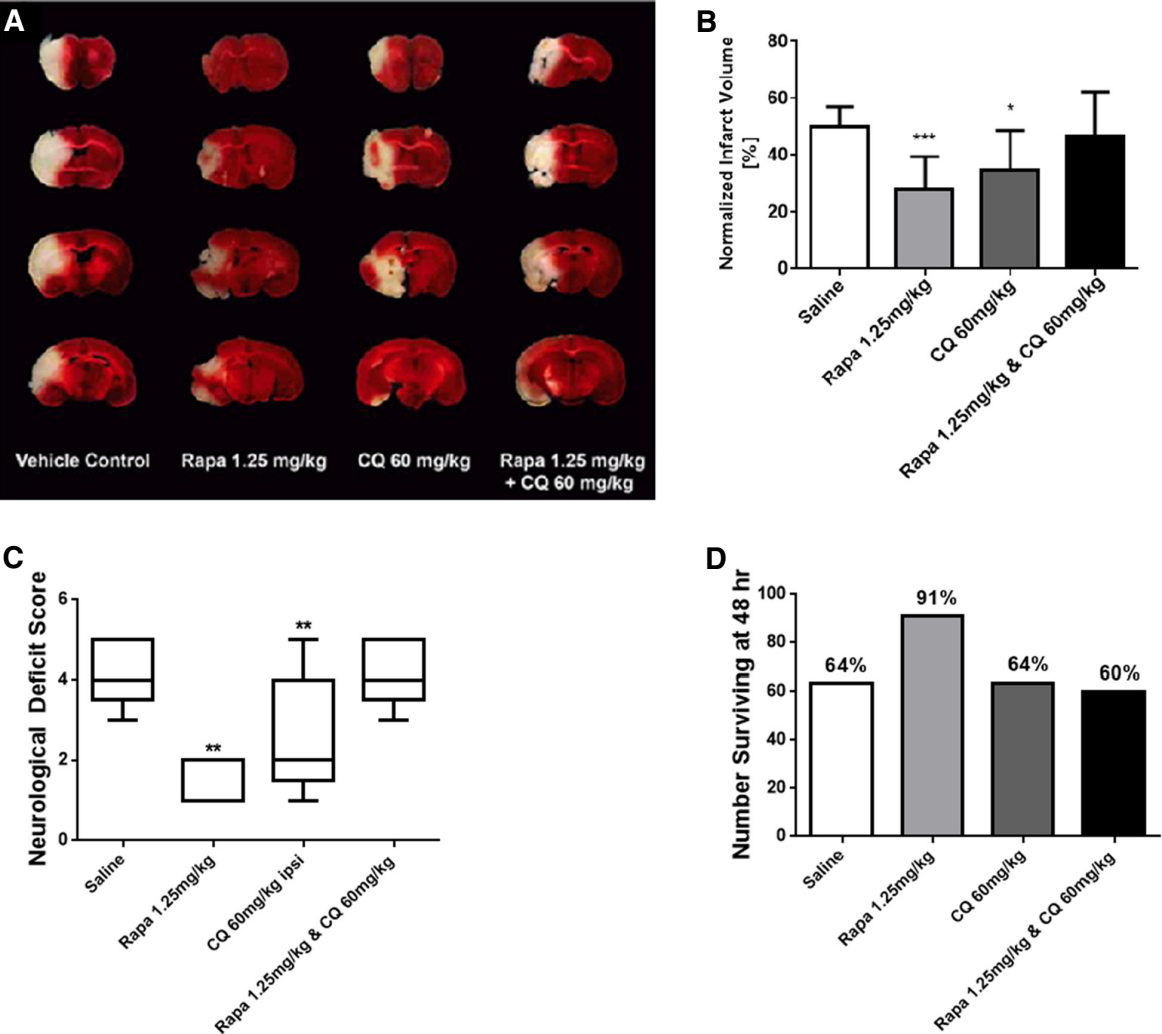
**Rapamycin improves infarct size**, **survival**, **and neurological outcomes after eMCAO.** The eMCAO study was performed with two independent sets of mice. In the first set there were 6 mice starting in the rapamycin, chloroquine and saline control groups they are included in the data in panels **B** &**D**, however, they were not assessed for behavior. The second set of mice had an n = 5 for each group, it also included a rapamycin and chloroquine dual treated group not included in the first set of mice. All of these mice received behavioral assessments and are included in the data for panels **B**, **C** &**D**. **A)**. Representative TCC stained brain sections 48 hours post-eMCAO stroke (n = 10 for rapamycin treatment, n = 7 for vehicle control and chloroquine treatments, and n = 3 for rapamycin and chloroquine dual treatment. These numbers represent the surviving animals at 48 hours out of eleven starting mice for the saline, chloroquine and rapamycin treated groups and five animals for the rapamycin and chloroquine dual treatment group and are the set of mice measured in panels **B** &**D**. **B)**. Normalized lesion volume at 48 hours post-eMCAO stroke. **C)**. Quantitation of the Bederson neurological deficit scores by treatment group (n = 5 out of 5 surviving mice for rapamycin treatment, n = 4 out of 5 surviving mice for chloroquine treatment, n = 3 out of 5 surviving animals for vehicle control and rapamycin and chloroquine dual treatment groups). **D)**. 48 hour post-eMCAO survival ratio (number of surviving mice vs total number of eMCAO stroked and treated mice) (n = 11 for vehicle control, chloroquine, and rapamycin treatments, and n = 5 for rapamycin and chloroquine dual treatment). Note tissue drop out in the lesion site is due to the fragility of the tissue, not surgical damage. (* = p < 0.05, ** = p < 0.01, *** = p < 0.005).

Next, we investigated the effect of pharmacologic modulation of autophagy in the eMCAO stroke model (Figure 3). Infarct analyses by TTC-staining after eMCAO revealed that rapamycin is more effective in attenuating the stroke injury lesion size (Figure 3A and 3B), in agreement with the MCAL model data (Figure 2). Rapamycin treatment significantly reduced the injury size compared to saline control (43.8% relative reduction; 28.1% ±11.3% versus 50.1% ±7.1%, P < 0.005). Chloroquine also significantly reduced the stroke injury (30.0% relative reduction; 35.0% ±13.8% versus 50.1% ±7.1%, P < 0.05), but to a lesser extent than rapamycin. In agreement with the results from MCAL experiments, the neuroprotection was lost in the combination treatment group of rapamycin and chloroquine (P = 0.0009 for the interaction; 46.8% ±15.6% versus 50.1% ±7.1% for saline P = 0.6).As shown in Figure 3C, rapamycin treatment improved the neurological deficit score compared to the saline treated control group (1.4 ± 0.5 versus 3.7 ± 0.6; P < 0.01). Chloroquine treatment also showed an improved neurological deficit score as compared to the saline treated control group (2.0 ± 0.8 versus 3.7 ± 0.6; P < 0.05), but less than that seen with rapamycin. When the two treatments were combined the improvements in neurological deficit score seen with the individual treatments, was lost (P = 0.0001 for the interaction; 3.7 ± 0.6 versus 3.7 ± 0.6, P = 1.0).The rapamycin only treatment seems most effective in improving survival post-stroke (Figure 3D). While not significant (P = 0.34), rapamycin treatment appears to increased survival at 48 hours post-stroke (10 out of 11 animals; ~91% survival) in comparison to the saline treated group (7 out of 11 animals survived; ~64% survival), chloroquine treatment (7 out of 11 animals survived; ~64% survival) or with the combined treatment group of rapamycin and chloroquine (3 out of 5 animals; ~60%).

## Discussion

Mizhuma et al., in a ground-breaking 2004 study [21], demonstrated high levels of LC3 isoforms, key markers of autophagy, in the brains of transgenic mice overexpressing a GFP-LC3 construct. The suggestion of high levels of LC3, and therefore autophagy, in the brain remained controversial for many years as research in neurodegenerative diseases revealed high levels of double membrane autophagosomes. This was incorrectly interpreted to conclude that the brain had a low level of autophagy, which is only up-regulated during neurodegeneration or injury [22-24]. In an effort to confirm if the brain has endogenously high levels of autophagy, we collected brain, heart, lungs, liver, kidney, spleen, and skeletal muscle, metabolically active tissues that have been thought to undergo active autophagy constitutively [25-27]. These were collected from 22–24 week old male C57BL6 mice and the levels of LC3I and II were assessed. We demonstrate that, relative to these tissues, brain expresses LC3I, the precursor to LC3II, and the active autophagic form, LC3II, at significantly higher levels. *In vitro* autophagic flux is a more reliable indicator of autophagic activity than static snap-shots of autophagic pathway markers, as is most commonly reported in the stroke literature [20,28]. We attempted to assess autophagic flux using a short-term *in-vivo* chloroquine treatment compared to untreated animals since there has not been a previous report of *in vivo* autophagic flux for murine brain. Chloroquine raises intracellular pH including lysosomal compartments thereby inhibiting acid-dependent lysosomal proteolytic degradation of cellular components within the autolysosome. This allows accumulation of autophagosomes and their contents including autophagy marker proteins. Moreover, when exposed to short-term autophagic inhibition these levels remain high. While the chloroquine treated brains do not have a statistically significant increase in LC3II, there is a strong trend supporting high autophagic flux, and importantly this is not abrogated by flux inhibition. If flux were low, or dysfunctional, there would be no change, or a decrease in LC3II, and potentially in LC3I, too. Future studies will examine optimizing *in vivo* inhibition of autophagosome turnover, both in terms of the inhibitor and its application, to assess *in vivo* autophagic flux. Importantly, the higher levels of both LC3I and LC3II suggest that brain has both the capacity for the initiation of higher levels of autophagy acutely in response to stress, as well as actually having a higher background constitutive autophagic flux. This suggests an enhanced ability to support constitutively active autophagy is critical for adult brain, possibly due to the high metabolic demand of the brain and lack of storage of nutrients like glucagon, which renders the brain, and neurons in particular, vulnerable to ischemic insult [29]. A high level of preformed autophagy constituents, (e.g. LC3I and II) ready to rapidly support autophagy, might provide an inherent degree of energy and nutrient buffering to the brain. Therefore, we hypothesized that driving autophagy is protective following stroke.

Autophagy has been well established to play a role in ischemic injury and neurodegeneration although the role(s) is complicated and not fully delineated [6,26,30]. For example, sustained stress may lead to depletion of key preformed autophagy pathway or regulatory proteins needed for successful protective autophagy [31-33]. Consequently, this may result in compromised or dysfunctional autophagy. While the role of autophagy in adult cerebral ischemia is still not clear, its induction appears to be protective and of potential translational value in limited slow reperfusion. However, whether autophagy is protective to neurons and other brain cells in response to ischemia may also be contextual, relative to the extent of ischemia and the presence, or type, of reperfusion. In earlier autophagy and stroke studies, tsMCAO with rapid complete reperfusion, was the most commonly used model. Importantly, the hyperemic effect of rapid and immediate complete reperfusion seen with this type of pre-clinical model is non-physiological relative to most human stroke. It can result in CBF rebounding above the pre-ischemic basal level. This effect may provide protection to both the ischemic core and penumbra when combined with pre-clinical treatments that reduce secondary injury, however, such treatments may not be protective with slow reperfusion [10]. Therefore, it may be important to find treatments that are protective when reperfusion is limited.

Ultimately this study supports the use of rapamycin, or analogues, as well as potentially other “pro-autophagic” approaches after stroke; specifically where there is partial recanalization and delayed reperfusion. Autophagy has recently been reported to potentially have two distinct roles during “ischemia” and “reperfusion” in studies of myocardial infarction [9]. Induction of autophagy provided protection during ischemia more than during reperfusion [34]. However, the timing of autophagy induction might be critical to obtain benefits during stroke. Therefore, future studies are still warranted. Importantly, given that the overwhelming majority of stroke patients do not have reperfusion reversals within the short time frame seen with tsMCAO, the increased vulnerability to secondary molecular cell death effects is not an issue. Indeed, this vulnerability may even be blocked by induction of active autophagy. Therefore, autophagy inducing interventions after stroke during the “golden hour” [10,12] may limit the expansion of penumbra and the necrosis of ischemic core. Rapamycin is a safe drug to use, possibly even in the presence of hemorrhagic stroke [35,36]. Therefore, rapid induction of autophagy, allowing neurons to survive loss of nutrients and energy production by using nutrient recycling and anaerobic ATP production may prevent, or significantly reduce, the perfusion/metabolism mismatch responsible for *primary* infarct expansion into the penumbra and beyond. This may allow a longer window of time for increased spontaneous recanalization based reperfusion and penumbral recovery, or even allow tPA use to be extended out in time and therefore to a larger percentage of the stroke patient population.

## Conclusion

By comparing both the enhancement and inhibition of autophagy side-by-side in two different pathophysiologically relevant models of ischemic injury, we were able to demonstrate that enhancement of autophagy by low dose rapamycin has advantages over its inhibition by chloroquine after stroke. Given rapamycin’s well-characterized safety profile, its use has significant translational potential, both due to its induction of autophagy, and even its secondary effects on inhibition of tissue destructive immune system responses. Based on the potential difference in response to autophagy regulation with rapid reperfusion in myocardial ischemic injury [9] it is important to determine if the positive effects seen in the eMCAO model on stroke injury outcomes are still present following rapid reperfusion with tPA. Since the eMCAO model used here is also suitable to utilize with IV-tPA thrombolysis, further post-eMCAO studies on the safety and benefits of either rapamycin, or chloroquine, as adjuvants to IV-tPA are warranted. Future studies will also address effects of age, gender and disease comorbidities (e.g. hypertension and diabetes) on stroke outcomes with modulation of autophagy.

## Competing interests

The authors declare that they have no competing interests.

## Authors’ contributions

KMB was involved in experimental design, experimental procedures and manuscript preparation. DLH, JRB, RK, HG, and GK were involved in experimental procedures. MHJ preformed statistical analysis. WDH, MNH, IYS, DCH, and PVS were involved in experimental design and manuscript review, in addition MNH preformed all stroke surgeries. SPT was involved in experimental procedures and manuscript discussion. All authors read and approved the final manuscript.

## Supplementary Material

Additional file 1: Figure S1 Determination of optimally protective dose for rapamycin and chloroquine in an MCAL mouse model of ischemic stroke injury. A). Representative anterior to posterior TCC stained brain sections 48 hours post-MCAL stroke treated with 40% DMSO (Vehicle Control), 1.25 mg/kg rapa, or 2.5 mg/kg rapa. Additionally a 0.625 mg/kg rapamycin group was assessed and was not different from the vehicle control (not shown) suggesting above 1.25 mg/K the protective effect was not enhanced. B). The 0.625 mg/kg rapa was comparable to DMSO control and was not repeated, both 1.25 mg/kg and 2/5 mg/kg were found to significantly reduce infarct size (p < 0.001), the lower dose of 1.25 mg/kg rapa was chosen to limit off target effects. C). Representative TCC stained brain sections 48 hours post-MCAL stroke treated with Saline (Vehicle Control), 30, 60, or 120 mg/kg CQ, D). The 30 and 120 mg/kg doses of CQ did not significantly improve infarct size, but the 60 mg/kg CQ did (p < 0.05) and was chosen for all other experiments. Click here for file
