# Supplementary figures and images for: Rapamycin up-regulation of autophagy reduces infarct size and improves outcomes in both permanent MCAL, and embolic MCAO, murine models of stroke
Source: Exp Transl Stroke Med. 2014 Jun 21;6:8. doi: 10.1186/2040-7378-6-8 (PMC4079187; doi:10.1186/2040-7378-6-8)

## Slide 1
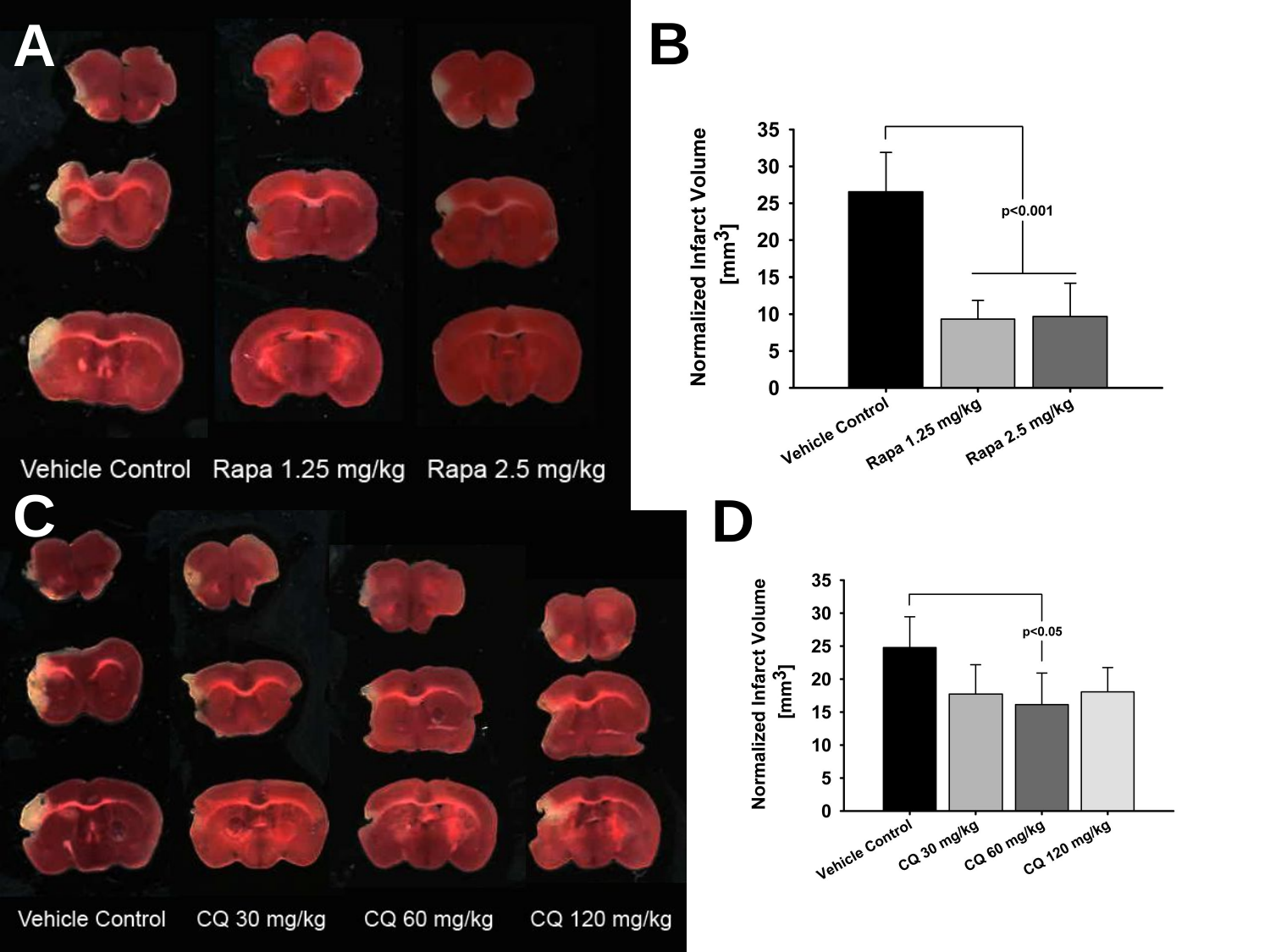

A
B
C
D

Supplement: Additional file 1: Figure S1 — Determination of optimally protective dose for rapamycin and chloroquine in an MCAL mouse model of ischemic stroke injury. A). Representative anterior to posterior TCC stained brain sections 48 hours post-MCAL stroke treated with 40% DMSO (Vehicle Control), 1.25 mg/kg rapa, or 2.5 mg/kg rapa. Additionally a 0.625 mg/kg rapamycin group was assessed and was not different from the vehicle control (not shown) suggesting above 1.25 mg/K the protective effect was not enhanced. B). The 0.625 mg/kg rapa was comparable to DMSO control and was not repeated, both 1.25 mg/kg and 2/5 mg/kg were found to significantly reduce infarct size (p < 0.001), the lower dose of 1.25 mg/kg rapa was chosen to limit off target effects. C). Representative TCC stained brain sections 48 hours post-MCAL stroke treated with Saline (Vehicle Control), 30, 60, or 120 mg/kg CQ, D). The 30 and 120 mg/kg doses of CQ did not significantly improve infarct size, but the 60 mg/kg CQ did (p < 0.05) and was chosen for all other experiments. [file 2040-7378-6-8-S1.ppt]
